# Perspective: Acknowledging complexity to advance the understanding of developmental coordination disorder

**DOI:** 10.3389/fnhum.2022.1082209

**Published:** 2023-01-06

**Authors:** Emily J. Meachon

**Affiliations:** Faculty of Psychology, University of Basel, Basel, Switzerland

**Keywords:** dyspraxia, complexity theories, complex adaptive systems, network analysis, research and practice

## Abstract

Developmental Coordination Disorder (DCD) is a heterogeneous neurodevelopmental disorder known for primary symptoms of motor learning and execution difficulties. Recent research has consistently suggested DCD symptoms span broadly beyond motor difficulties, yet a majority of research and practice approaches the investigation, diagnosis, and treatment of DCD with a reductionist framework. Therefore, this paper suggests the paradigm of complexity theory as a means for better conceptualization, assessment, and treatment of DCD. First, the perspective of complexity theory and its relevance to DCD is described. Then, examples from recent research which attempt to acknowledge and capture the complex nature of DCD are highlighted. Finally, suggestions for considering and measuring complexity of DCD in future research and practice are provided. Overall, the perspective of complexity can propel the research forward and improve the understanding of DCD relevant to assessment and treatment. The complexity paradigm is highly relevant to describing the evolving and multidimensional picture of DCD, understanding heterogeneous symptom profiles, making connections to interconnected secondary symptoms, and beyond.

## Introduction

Developmental Coordination Disorder (DCD) is a neurodevelopmental disorder characterized by difficulties in motor learning, execution, and coordination (American Psychiatric Association, 2013; Blank et al., 2019) with many secondary symptoms (Kirby et al., 2013; Leonard and Hill, 2015; Bernardi et al., 2018; Tal Saban and Kirby, 2018; Zwicker et al., 2018; Sartori et al., 2020; Meachon et al., 2022b). General knowledge surrounding secondary difficulties in DCD, such as differences in executive functioning, remains limited and under examined (Fogel et al., 2021), especially compared to similar neurodevelopmental disorders such as Attention-Deficit/Hyperactivity Disorder (ADHD; Meachon et al., 2022b). This can be partially attributed to clinicians and researchers following the narrow, reductive diagnostic criteria of DCD which exclusively considers motor symptoms (e.g., DSM-5: American Psychiatric Association, 2013; Purcell et al., 2015). In addition, consensus on the relevance of secondary concerns, such as executive functioning difficulties, in DCD is relatively new (Leonard and Hill, 2015; Blank et al., 2019) and may not yet be integrated with multifaceted screening approaches among all clinicians and researchers examining DCD. However, this discrepancy can have major consequences on understanding DCD and its secondary symptoms. It is imperative to resolve these issues in order to provide more accurate detection, diagnosis, and support for individuals with DCD. This is a challenge in modern research and clinical practice given that DCD is a highly complex condition (Blank et al., 2019) and motor systems have substantial interactions to cognitive systems (i.e., executive functioning, intelligence) that are not easily disentangled (Diamond, 2000; Asonitou et al., 2012; Schott et al., 2016).

To advance the understanding of DCD, researchers and practitioners must consider the complexity of DCD. Therefore, this perspective paper makes a case for the application of complexity theory to view DCD from several angles. First, complexity theory is defined. Next, the perspective of complexity is considered when viewing the motor system in DCD and in general. Then, secondary concerns of DCD are reviewed as further examples of complexity in the motor system and beyond. Additionally, advances in recent DCD research which have considered complexity are highlighted. Finally, suggestions for acknowledging complexity in research, assessment, and support for DCD are provided.

### Defining complexity in DCD

To apply the perspective of complexity to DCD, the key aspects of this paradigm must first be defined. A complex system is one which is (1) non-linear, (2) self-organizes, and (3) has emergent properties (Holland, 2014). This means that, first, a complex system cannot be defined by linear parameters, or a simple input to output equation. Second, complex systems create their own form of order, or self-organize, without external influence (Green et al., 2019). Third, a complex system has emergence, or features that equal more than the sum of its parts. When emergence takes place, it is often when something unexpectedly and suddenly appears in a complex system (Galatzer-Levy, 2002). One example of a complex biological system is the brain, a system with many nodes (e.g., neurons), which forms self-organized networks that work together in a decentralized manner, and has emergent characteristics not inherently obvious from observing the system’s parts alone (e.g., consciousness; Bassett and Gazzaniga, 2011; Holland, 2014; Mastrandrea et al., 2017). These properties make complex systems challenging to predict and sometimes difficult to conceptualize.

Generally, a perspective of complexity considers broad-scale interactions within a system and a degree of uncertainty as opposed to a reductionist approach which suggests complex systems can be explained in entirety. The complexity framework can be particularly important for viewing mental or physical health, where reductionist approaches might harm patients (e.g., assuming symptoms are isolated to one condition), or prevent progression in research (e.g., assuming all elements of a system have been considered). Therefore, it is important to consider the complexity of DCD symptoms.

### Complexity of motor and cognitive symptoms of DCD

The primary symptoms of DCD are motor-based, including difficulties with acquiring and executing fine and gross motor functions as well as general coordination (American Psychiatric Association, 2013). However, the primary symptoms substantially vary from one individual with DCD to the another. As such, even though motor symptoms are the most understood aspects of DCD (Blank et al., 2019), there is still some ambiguity in defining the motor profile of DCD. For example, studies which have aimed to identify subtypes of DCD based on motor symptoms yielded inconsistent results regarding the number of subtypes that existed and the major motor difficulties common to each subtype (Vaivre-Douret et al., 2011; Lust et al., 2022). More specifically, Lust et al. (2022) identified four major subtypes (all reduced motor skills, reduced motor skills except for gross motor skills, gross motor/balance difficulties, fine motor difficulties), while Vaivre-Douret et al. (2011) found three subtypes (ideomotor dyspraxia, visual spatial and visual constructional dyspraxia, mix dyspraxia). When considering additional cognitive symptoms, Asonitou et al. (2022) identified six clusters based on motor and cognitive assessment among students with and without DCD. Therefore, it is likely there is substantial complexity in the motor symptoms of DCD and the related motor system, which only increases when considering cognitive interactions.

At the basis of motor development, there are fundamental connections made between the motor system and other networks in the brain, including cognitive systems responsible for higher-order processing such as executive functioning, attention, and more (Diamond, 2000; Raz, 2004; Mendoza and Merchant, 2014; Leisman et al., 2016). These symptoms develop in parallel and are highly interconnected through the lifespan, observable at both the neural and behavioral levels (e.g., Diamond, 2000; McLeod et al., 2016; Schott et al., 2016). For example, Piek et al. (2008) used trajectory analysis to show gross motor ability at a young age can predict cognitive ability at a later age. This indicated that it may be possible to predict future cognitive delays and manage them sooner in parallel to motor support (Piek et al., 2008). In addition, the executive functions of working memory and task-switching can be recruited during walking among typically developing children (Möhring et al., 2020), indicating that the task of walking alone is not exclusive to the motor system. However, it is not yet clear if cognitive resources are allocated in order to walk, or as a product of walking (Leisman et al., 2016).

Motor and cognitive overlap could explain why some cognitive abilities, such as intelligence, differ among individuals with DCD compared to their typically developing peers (e.g., Jaščenoka and Petermann, 2018; Jascenoka and Walter, 2022). Especially considering that DCD is based in motor difficulties and the role of cognitive impairment is heavily debated (Fogel et al., 2021) but highly relevant (Purcell et al., 2015). Another category of cognition known as executive functioning, or regulating and controlling higher-order cognitive processes including inhibition, working-memory, and task-switching, is commonly reduced in DCD (Leonard and Hill, 2015; Bernardi et al., 2018; Sartori et al., 2020; Fogel et al., 2021). Executive functioning difficulties observed in DCD, are often thought to be caused by co-occurring ADHD (Farran et al., 2020). However, even when individuals with co-occurring ADHD are removed from samples, studies have still identified reduced cognitive performance in DCD compared to typically developing individuals (e.g., Licari et al., 2015; Manicolo et al., 2017; Klupp et al., 2021). In addition, adults with DCD often report executive functioning difficulties to be one of their most prominent concerns, with greater relevance than motor difficulties (Purcell et al., 2015). In this study, executive functioning difficulties were classified from free-text responses related to trouble with organizational skills, memory, planning ahead and prioritizing, and speed of processing (Purcell et al., 2015). Therefore, there seems to be a high relevance of executive functioning difficulties in DCD. However, it remains unclear if cognitive difficulties can be explained by co-occurrence, cognitive-motor interference, or are present in DCD as secondary symptoms.

### Key secondary concerns in DCD

There is considerable heterogeneity in the primary and secondary symptoms of DCD at all ages (Zwicker et al., 2018; Blank et al., 2019) including secondary psycho-social (e.g., Kirby et al., 2013; Draghi et al., 2020) and cognitive concerns (Purcell et al., 2015; Fogel et al., 2021). To date, there are no diagnostic suggestions regarding executive functioning in DCD, however, there are general guidelines for intellectual impairment. According to the latest guidelines in the DSM-5, DCD should not be diagnosed if an intellectual disability is present (DSM-5, American Psychiatric Association, 2013). Nonetheless, children with DCD can have significantly lower IQ scores compared to their peers (Jaščenoka and Petermann, 2018; Jascenoka and Walter, 2022). Therefore, in a recent international expert consensus on DCD, it was suggested that below-threshold IQ scores should not prevent a DCD diagnosis (Blank et al., 2019). Furthermore, it has been observed that motor skills and intelligence are highly related (Piek et al., 2008; Klupp et al., 2021) and motor outcomes can be partially explained by reductions in IQ scores (Smits-Engelsman and Hill, 2012). This aligns with the theory that a deficit in the cognitive system can adversely impact the interconnected motor system, and vice versa (Diamond, 2000).

Another area of concern is the tendency for individuals with DCD to have more anxiety and depressive symptoms than their typically developing peers (Kirby et al., 2013; Rigoli and Piek, 2016; Draghi et al., 2020; Harris et al., 2021). It is suspected that these concerns might be a product of motor difficulties, such that negative experiences from motor symptoms can lead to reductions in self-perceived competence and decreases in physical activity participation (Cairney et al., 2013; Batey et al., 2014). This has the potential to become a vicious circle for those with DCD, in which physical activity is avoided for fear of judgment, and thereby, reductions in exercise lower one’s motor skills and mental health (Wipfli et al., 2011; Cairney et al., 2013; Holfelder and Schott, 2014). However, more research is needed to understand these links (Harris et al., 2022). Screening for depression and anxiety is not presently part of DCD assessment, but should be considered for more adequate psychological and social support (Meachon et al., 2022b).

Importantly, secondary symptoms and concerns of patients can vary widely between individuals with DCD. They can also change over the course of an individual’s lifetime and shift due to learned compensatory behaviors developed on an individual basis and co-occurrences (Wilmut, 2017; Cignetti et al., 2018). Notably, co-occurrence is the rule rather than the exception for DCD and other neurodevelopmental disorders (Cleaton and Kirby, 2018). Co-occurrence can be substantial and add to the challenges of disentangling motor and cognitive processes in DCD. For example, DCD and ADHD are reported to co-occur in up to 50% of cases (Blank et al., 2019) and have numerous overlapping motor and executive functioning symptoms (Kaiser et al., 2015; Meachon et al., 2022a). DCD and ADHD are separate conditions, which both have motor and cognitive difficulties compared to typically developing peers, but may still have their own motor and cognitive profiles (Goulardins et al., 2015; Meachon et al., 2022a). Therefore, understanding the nature of motor and cognitive overlaps has the potential to support disentangling DCD and ADHD symptoms and improving differential diagnosis. Overall, it may prove useful to examine neurodevelopmental disorders in a holistic format, screening for more than just the suspected condition(s) (e.g., Lange, 2018). This approach would also support capturing more informative individual differences and unique adaptations to DCD symptoms.

### Recent advancements in DCD research

There are some approaches which have acknowledged the heterogeneity and complexity in DCD which serve as key examples of propelling the understanding of DCD forward. The network approach could be used to acknowledge DCD symptoms with a more detailed picture than traditional single-symptom investigations (Fulceri et al., 2019). However, network approaches that describe any aspect of DCD are limited in number to date. Those which have considered such an approach have focused exclusively on brain connectivity (e.g., McLeod et al., 2014, 2016), which is distinct from a methodological network analysis but still takes a holistic approach to understanding brain function and structure. For example, several studies have investigated resting state fMRI connectivity between children with DCD and/or ADHD, identifying notable distinctions in the corpus callosum between children with DCD versus ADHD (Langevin et al., 2014a; McLeod et al., 2014; Rohr et al., 2021). Unique structural differences have also been noted between those with DCD, ADHD, and both conditions, with more regions implicated in co-occurring cases (Langevin et al., 2014a,b) and reduced responsiveness to intervention (Izadi-Najafabadi et al., 2022). This signifies that individuals with co-occurring DCD and ADHD have a unique, and likely more severe, symptomatic profile compared to those with just DCD or ADHD. This pattern has been noted in other studies of brain structure and function (e.g., McLeod et al., 2016) as well as studies of related outcomes between these groups (e.g., Meachon and Alpers, 2022).

Furthermore, studies examining DCD compared to typically developing groups have noted dysfunction in attentional networks (Querne et al., 2008), unique activation during fine motor tasks (Zwicker et al., 2010), and reduced activation in areas associated with working memory and motor imagery during visuospatial tasks (Licari et al., 2015), among other important findings (for complete reviews of neural aspects of DCD see Brown-Lum and Zwicker, 2015; Biotteau et al., 2016; Dewey and Bernier, 2016; Fuelscher et al., 2018; Hyde et al., 2019). It is also possible to observe network alterations among those with DCD in response to effective interventions such as exercise-based interventions (e.g., Tsai et al., 2012), standard motor skill intervention (e.g., CO-OP; Izadi-Najafabadi et al., 2022), and combined action observation and motor imagery training (Scott et al., 2021). Continuing to build on evidence for between-group structural and functional differences could enable future research to create precise predictive models for neural differences distinctly observed in DCD (Mäki-Marttunen et al., 2019).

Another example of considering complexity in DCD can be seen in studies which represent co-occurrences such as ADHD and ASD. For example, the inclusion of comparisons of single-occurring, co-occurring, and typically developing participants can be implemented in order to identify if certain overlapping symptoms, such as cognitive and motor difficulties, are due to co-occurrence or inherent difficulties in each condition (e.g., Cignetti et al., 2018; Meachon et al., 2021; Rohr et al., 2021). While the general classification into groups may be considered reductive on some levels, comparing multiple clinical groups is a more representative approach to the common comparison of a DCD group to a typically developing group. The multi-group and comorbidity-based approach has been suggested as a fundamental step toward identifying motor performance differences inherent to each of the developmental disorders (Cignetti et al., 2018).

Another important approach is the integration of longitudinal designs in measuring motor and cognitive changes overtime (e.g., Wilson et al., 2020; Landgren et al., 2021). Such approaches have already identified persistent executive functioning difficulties in DCD in childhood (Wilson et al., 2020) and more negative psycho-social, health, and employment outcomes into adulthood (Landgren et al., 2021).

In addition, studies which utilize neurofeedback for continuous adaptive performance corrections integrate a top-down approach acknowledging the complexity of DCD (e.g., Cheng et al., 2022). One example identified that individuals with DCD were not able to make adaptive postural compensations when faced with an unexpected inclination (Cheng et al., 2022). This approach is useful to inform the state of motor ability in a specific domain, and as a means for future training to improve this difficulty in individuals with DCD.

## Discussion

Developmental Coordination Disorder is a complex neurodevelopmental disorder that has consequences for the motor system and many other domains. To date, considerations of complexity have supported an enhanced understanding of DCD and have the potential to substantially improve the future research and clinical picture of numerous aspects of DCD, including motor symptoms, secondary problems, and more. It is important to consider the complex and interconnected nature of the motor system in DCD and related primary and secondary symptoms.

### Complexity in primary and secondary symptoms of DCD

While there is no doubt DCD involves substantial motor learning and execution difficulties, there are still aspects of the motor symptoms which are not yet fully understood. This is evident through a lack of consensus on the potential for and nature of DCD subtypes (Vaivre-Douret et al., 2011; Lust et al., 2022), which becomes even more complex when considering additional subtypes for motor and cognitive skills (Asonitou et al., 2022). Furthermore, the motor system does not act alone, often integrating cognitive resources such as executive functions (Diamond, 2000; Asonitou et al., 2012; Leisman et al., 2016; Möhring et al., 2020). Therefore, it can be a challenge to isolate and describe motor symptoms of DCD, as they often are closely intertwined with high order cognitive processes.

It has become increasingly clear in recent research that DCD is not limited to motor symptoms (e.g., Kirby et al., 2013; Leonard and Hill, 2015; Purcell et al., 2015; Bernardi et al., 2018; Blank et al., 2019; Draghi et al., 2020; Sartori et al., 2020), in part, due to the interconnected nature of the motor system. It has been suggested that executive functions could be a key component of DCD especially among adults (e.g., Purcell et al., 2015; Meachon et al., 2022a). However, there are mixed results regarding the extent to which executive functions can be considered core difficulties of DCD (Fogel et al., 2021). One possibility for this ambiguity could be the inconsistency of measurement techniques used to measure executive functions in DCD, and in general. For example, two different validated tasks that both measure inhibition can engage different mental and physical resources and, ultimately, measure unique processes (Miyake et al., 2000). Furthermore, all types of executive functioning tasks typically involve a motor or verbal response, which make motor and cognitive contributions to performance difficult to disentangle. Additionally, accuracy and reaction time on executive functioning tasks can be reduced among individuals with motor difficulties from the motor contribution alone, but would be interpreted as a cognitive deficit (Meachon et al., 2021). Disentangling executive functions from motor functions has been attempted in some methodological approaches, e.g., diffusion modeling of reaction times (Karalunas and Huang-Pollock, 2013; Ratcliff et al., 2016) and research paradigms, e.g., dual-task (Al-Yahya et al., 2011). However, there are still overlaps in motor and cognitive processes that cannot be corrected, and controlling for these overlaps remains a challenge in modern research.

One way to deal with the complexity and substantial overlaps between executive and motor functions is to build treatment approaches for symptoms and support for individuals with DCD that view the symptomatic system at the macro-level, or in a top-down manner (Fogel et al., 2021). With this approach, the precise connections and mechanisms of motor and cognitive systems are not considered at the first step and, to a certain degree, accepted as phenomena in a “black box.” Examples from recent research show this approach can be beneficial to understanding DCD (e.g., Cheng et al., 2022). Therefore, it is important to determine the etiology and mechanisms of DCD, but equally important and relevant to work with the complexity of DCD before these factors are fully described.

### Future directions in research

A complexity perspective might suggest considering the strength of cognitive and motor links in a network relationship compared to other engaged systems in a given task (e.g., language), rather than attempting to isolate motor and cognitive systems from each other. This application of network models to describe symptoms can support early identification of mental health conditions and co-occurrences as they emerge (Fried et al., 2017). This could improve the support system for patients by signaling a change is needed, such as including a psychotherapist in treatment if signs of co-occurring depression arise.

Similarly, major categories of symptoms and secondary problems can be depicted across a broad spectrum using the Research Domain Criteria for DCD, or neurodevelopmental disorders in general (RDoC; Mittal and Wakschlag, 2017). This framework involves screening for symptoms in many different domains (e.g., motor, cognitive, emotional, social, mood, etc.). The RDoC has already been successfully applied to other neurodevelopmental disorders, such as autism spectrum disorder (ASD; Harrison et al., 2021) and recommended for other conditions such as ADHD (Heidbreder, 2015). Such multidimensional approaches provide a unique solution to capturing the numerous primary and secondary symptoms of conditions such as DCD, particularly when the network is examined more than once to detect key changes over time. There is a clear need for more network approaches to understand DCD, and this approach is considered essential in future investigations of developmental disorders (e.g., Fulceri et al., 2019; Nicolson and Fawcett, 2019). The DCD research community can look to existing network classifications of ASD symptoms and co-occurrences in networks as examples (e.g., Fulceri et al., 2019; Kelmanson, 2019).

### Acknowledging complexity in clinical practice

There are numerous ways the complexity of DCD can be acknowledged in clinical practice. For one, the motor, cognitive and other secondary symptoms could be assessed as a standard to understand the full picture of a patient’s experience and provide better support (Barnett, 2014). A holistic approach to diagnosis should also involve in-depth screening for all potential co-occurring neurodevelopmental and mood disorders. Beyond this, a broad range of diagnostic tools must continue to be developed in order to prevent a single measure becoming a gold standard (Barnett, 2014). This practice would help encourage and sustain gathering a well-rounded perspective of patient’s strengths and difficulties in motor and non-motor realms (Barnett, 2014).

Notably a multidimensional screening approach (e.g., RDoC; Mittal and Wakschlag, 2017) may prove beneficial for understanding the complex symptoms across the neurodevelopmental conditions in research, but should be applied with caution in clinical practice. This approach would uphold the perspective of complexity and could be useful to gather clinical information, but it is crucial that it does not fully replace classification of conditions upon which diagnosis, support, and funding for patients are based.

Regarding treatment, dynamic communication and adaptive support through multidisciplinary collaboration with clinicians of different fields could be extremely beneficial to individuals with DCD and those with other neurodevelopmental disorders (Rutherford et al., 2021). This approach could enable experts from different fields to contribute unique expertise toward each of the varied difficulties present in DCD across motor, cognitive, and secondary symptoms. This would acknowledge the complexity of DCD and, in turn, strengthen the understanding of DCD. Such an approach has been exemplified by Beaudry-Bellefeuille et al. (2021) who mapped out non-hierarchical relationships between the patient and their family and the clinicians involved in supporting specific problems in children with ASD.

### Limitations and the reductionist perspective

It is common for DCD and related research methodology to function in a reductionist framework. To some degree, this approach is necessary in order to cope with the reductive nature of existing diagnostic criteria for DCD (Purcell et al., 2015). It is also common for grant agencies and journals to prefer reductionist approaches, and thereby, limiting some research to this framework. Nonetheless, the reductionist approach has merit, often leading to important scientific insights from the bottom-up, and has supported the foundational research and understanding of DCD thus far. The use of reductionist approaches should not be brought to a full stop, but it is essential that future research gives more consideration to the perspective of complexity instead, or in parallel. Given the numerous forms in which complexity can be observed in DCD, the integration of a new paradigm provides a major path forward.

## Conclusion

Overall, promising strides have been made to build a clearer picture of DCD. The perspective of complexity can support advancements in the classification, assessment and treatment of DCD and is a key paradigm for research to consider in order to advance the general understanding of DCD.

## Data availability statement

The original contributions presented in this study are included in this article/supplementary material, further inquiries can be directed to the corresponding author.

## Author contributions

EJM was responsible for all aspects of the article, including literature searches, theorizing, preparation and writing the manuscript.
